# Transcriptomic and metabolomic integration as a resource in grapevine to study fruit metabolite quality traits

**DOI:** 10.3389/fpls.2022.937927

**Published:** 2022-10-20

**Authors:** Stefania Savoi, Antonio Santiago, Luis Orduña, José Tomás Matus

**Affiliations:** ^1^ Department of Agricultural, Forest and Food Sciences, University of Turin, Grugliasco, Italy; ^2^ Institute for Integrative Systems Biology (I2SysBio), Universitat de València-CSIC, Paterna, Spain

**Keywords:** omics integration, Vitviz, database, integrape, *Vitis vinifera*

## Abstract

Transcriptomics and metabolomics are methodologies being increasingly chosen to perform molecular studies in grapevine (*Vitis vinifera* L.), focusing either on plant and fruit development or on interaction with abiotic or biotic factors. Currently, the integration of these approaches has become of utmost relevance when studying key plant physiological and metabolic processes. The results from these analyses can undoubtedly be incorporated in breeding programs whereby genes associated with better fruit quality (e.g., those enhancing the accumulation of health-promoting compounds) or with stress resistance (e.g., those regulating beneficial responses to environmental transition) can be used as selection markers in crop improvement programs. Despite the vast amount of data being generated, integrative transcriptome/metabolome meta-analyses (i.e., the joint analysis of several studies) have not yet been fully accomplished in this species, mainly due to particular specificities of metabolomic studies, such as differences in data acquisition (i.e., different compounds being investigated), unappropriated and unstandardized metadata, or simply no deposition of data in public repositories. These meta-analyses require a high computational capacity for data mining *a priori*, but they also need appropriate tools to explore and visualize the integrated results. This perspective article explores the universe of omics studies conducted in *V. vinifera*, focusing on fruit-transcriptome and metabolome analyses as leading approaches to understand berry physiology, secondary metabolism, and quality. Moreover, we show how omics data can be integrated in a simple format and offered to the research community as a web resource, giving the chance to inspect potential gene-*to*-gene and gene-*to*-metabolite relationships that can later be tested in hypothesis-driven research. In the frame of the activities promoted by the COST Action CA17111 INTEGRAPE, we present the first grapevine transcriptomic and metabolomic integrated database (TransMetaDb) developed within the Vitis Visualization (VitViz) platform (https://tomsbiolab.com/vitviz). This tool also enables the user to conduct and explore meta-analyses utilizing different experiments, therefore hopefully motivating the community to generate Findable, Accessible, Interoperable and Reusable (F.A.I.R.) data to be included in the future.

## Introduction

**Figure 1:**
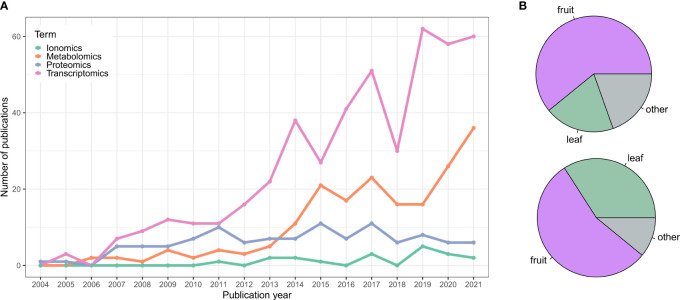
**(A)** Publication trend of grapevine omics studies. Data was collected from NCBI using different queries and keywords for each category to be as comprehensive as possible and retrieve most of the works. Transcriptomics query: 1) RNA-Seq (excluding microarray data): (transcriptom*[Title/Abstract] OR transcript profiling[Title/Abstract] OR mRNA expression[Title/Abstract] OR RNA-Seq[Title/Abstract] OR RNASeq[Title/Abstract] OR RNA Sequencing[Title/Abstract] OR RNA-Sequencing[Title/Abstract]) AND (grapevine[Title/Abstract] OR grape[Title/Abstract] OR Vitis[Title/Abstract] OR V. vinifera[Title/Abstract]). 2) Microarray: (microarray[Title/Abstract] OR Affymetrix[Title/Abstract] OR CombiMatrix[Title/Abstract] OR NimbleGen[Title/Abstract]) AND (grapevine[Title/Abstract] OR grape[Title/Abstract] OR Vitis[Title/Abstract] OR V. vinifera[Title/Abstract]). Proteomics query: (proteom*[Title/Abstract] OR protein profiling[Title/Abstract]) AND (grapevine[Title/Abstract] OR grape[Title/Abstract] OR Vitis[Title/Abstract] OR V. vinifera[Title/Abstract]). Metabolomics query: (metabolom*[Title/Abstract] OR metabolite profiling[Title/Abstract] OR metabolite analyses[Title/Abstract] OR metabolic response[Title/Abstract]) AND (grapevine[Title/Abstract] OR grape[Title/Abstract] OR Vitis[Title/Abstract] OR V. vinifera[Title/Abstract]). Ionomics query: (ionom*[Title/Abstract] OR elemental profiling[Title/Abstract] OR mineral elements[Title/Abstract] OR cations[Title/Abstract]) AND (grapevine[Title/Abstract] OR grape[Title/Abstract] OR Vitis[Title/Abstract] OR V. vinifera[Title/Abstract]). Only the studies published until 12/2021 were considered. Data were manually curated to remove outliers. **(B)** Pie chart showing the percentage of transcriptomics (*top panel*) and metabolomics (*bottom panel*) studies focusing on fruit tissues, leaves or other grapevine organs.

There has been more than 15 years of omics studies accumulated in grapevine (*Vitis vinifera* L.) to understand processes related to its development and interaction with the environment. The vast amount of data generated has allowed us to understand this species singularity in terms of its adaptive traits, but has also permitted us to explore its diversity, especially related to plant performance (e.g., stress resistance, vigor, yield, etc.) and fruit quality. This data includes genome assemblies of many different cultivars and clones of *V. vinifera* and their wild American or Asian-related species and, in decreasing order of the number of published studies, transcriptomic, metabolomic, proteomic, and ionomic data (**Figure 1** and **Supplementary Table 1**).

A significant achievement in the grapevine omics era was the release of two independent genome sequences in 2007, with one being accomplished by a French-Italian Public Consortium (Jaillon et al., 2007), and the second through an Italian-American Collaboration (Velasco et al., 2007). Grapevine then became the first fruit genome to be sequenced and the fourth among plants after *Arabidopsis thaliana* (Arabidopsis Genome Initiative, 2000), rice [*Oryza sativa* subsp. indica and subsp. japonica (Goff et al., 2002; Yu et al., 2002)] and poplar (*Populus trichocarpa*) (Tuskan et al., 2006). Since then, the grapevine community has witnessed an increase of high-throughput next-generation sequencing techniques (e.g., long-read sequencing of RNA and DNA) and the availability of updated grapevine genome assemblies of the reference PN40024 (Canaguier et al., 2017; unpublished data), with updated gene functional annotations incorporated in the recently developed Grape Gene Reference Catalogue (Navarro-Payá et al., 2022). We have observed a cascade of genome sequences of *V. vinifera* top-quality wine-making cultivars, and genomes of *Vitis* species important for breeding purposes (da Silva et al., 2013; Venturini et al., 2013; Chin et al., 2016; Minio et al., 2019a; Minio et al., 2019b Vondras et al., 2019; Zhou et al., 2019; Massonnet et al., 2020; Magris et al., 2021). In parallel with genomic/transcriptomic advances, the technological improvement of analytical techniques such as high-resolution liquid and gas chromatography coupled to mass spectrometry, in terms of sensitivity, accuracy, and resolution, has led to a massive amount of metabolomic data from heterogeneous experimental designs, many of which are not public to date.

Comprehensive studies pointing to the expression of the transcriptome or the abundance of the so-far known grape metabolites have boosted the understanding of grapevine physiology in the context of crop and fruit improvement. However, data/metadata interpretation generally encounters difficulties of reusability, for example in meta-analysis studies, either due to high variability and heterogeneity of the associated data or to the presence of partial, misleading, or incomplete experimental descriptions. These descriptors should typically include detailed information on the experimental set-up, report the plant materials used and adopt standardized cultivar names, organs, and developmental stages. Although this comprehensive praxis is highly recommended, in most cases, we notice that even raw data is not entirely available in public databases. Different guidelines have been generated to fill this gap, focusing on harmonizing plant and experiment descriptors. These include standard ontologies, lists of tools, and systematic information for describing omics analyses and tutorials for data submission in public repositories. These guidelines have been recently adopted by the viti-oenology community, sponsored by the COST Action CA17111 INTEGRAPE, in order to adhere to the *findable*, *accessible*, *interoperable* and *reusable* (F.A.I.R) principles (Wilkinson et al., 2016). A section specifically related to grape and wine metabolomics has been recently published (Savoi et al., 2021a) to encourage the grapevine community to follow these guidelines and share metabolomic data in open repositories such as MetaboLights (Haug et al., 2020). These efforts were made because, contrary to the wave of transcriptomic data available online, unfortunately, only very few metabolomic studies are so far accessible to the public.

## History of omics studies in grapevine

### Transcriptomics

The first records of high-throughput grapevine transcriptomic studies date back to 2005, exploring the changes in gene expression during berry development. Terrier et al. (2005) generated 50-mers oligoarrays bearing a set of approximately 3,200 unigenes from *Vitis vinifera* from nine berry developmental stages to provide the first global picture of the genetic program of grape berry development. The authors were able to discriminate differences in gene expression between hard green and soft green berries at the onset of ripening (veraison), pointing out that remarkable changes may occur within a short period and that 25% of the transcripts were significantly activated or repressed between the green and the ripening phases. In a second study (da Silva et al., 2005), the authors retrieved all the available *Vitis* sequences deposited in GenBank representing numerous *Vitis* species, cultivars, organs, plant developmental stages, and stress treatments. The analysis concluded that each stage of development was characterized by distinct gene expression patterns, including numerous stage-specific transcripts. Interestingly, they identified a MADS-box gene as a putative regulator of grape berry development. A third study (Waters et al., 2005) used the same technology to explore gene expression patterns throughout grape berry development revealing sets of genes with distinctive or similar expression profiles over the course of berry development. Finally, another pioneering study (Grimplet et al., 2007) used a brand new Affymetrix GeneChip^®^ *Vitis vinifera* oligonucleotide microarray to study tissue-specific mRNA expression in berry skin, flesh, and seeds in well-watered and water deficit plants at fruit maturity, listing a repertoire of expressed genes, highlighting those modulated by drought stress. In particular, stress modulated around 13% of the genes, mainly in the pulp and skin. In synthesis, these groundbreaking studies paved the way for future grapevine transcriptomic works developing compendiums of gene expression, studying gene profiles during grape berry development and setting the first milestone in understanding grapevine physiology.

The first grapevine study that specifically employed RNA sequencing using the Illumina platform corresponds to that of Zenoni et al. (2010). The authors focused on three stages of berry development indicated as post-setting, veraison, and ripening, to gain insight into the wide range of transcriptional responses associated with the development of fruits. They detected the expression of 17,324 genes during berry development, of which 6,695 were expressed in a stage-specific manner. Moreover, they identified alternative splicing events for 385 genes suggesting a considerable transcript complexity in developing berries. The grapevine expression atlas of the cultivar (cv.) ‘Corvina’ (Fasoli et al., 2012) was later released based on the Nimblegen microarray platform (29,000 genes spotted) representing the first wide study of global gene expression in a complete repertoire of grape organs, including 54 reproductive and vegetative tissues at different developmental stages. This work highlighted a fundamental transcriptome reprogramming during maturation with the activation of a mature/woody developmental program, which was mainly inactive during the vegetative/green stage. Subsequent studies also focused on berry development, ripening, and post-harvest of different cultivars (Pilati et al., 2007; Lijavetzky et al., 2012; Sweetman et al., 2012; Cramer et al., 2014; Gouthu et al., 2014; Pilati et al., 2014; Guo et al., 2016; Zenoni et al., 2016; Ghan et al., 2017; Massonnet et al., 2017; Shangguan et al., 2017; Balic et al., 2018; Fasoli et al., 2018; Savoi et al., 2019; Cramer et al., 2020; Guo et al., 2020; Savoi et al., 2021b; Theine et al., 2021). At the same time, other studies considered the development of tendrils and inflorescences (Díaz-Riquelme et al., 2014), buds (Díaz-Riquelme et al., 2012; Pucker et al., 2020; Shangguan et al., 2020), flower (Sreekantan et al., 2010; Grimplet et al., 2017; Vannozzi et al., 2021), leaf (Pervaiz et al., 2016), fruits of seeded/seedless cultivars (Nwafor et al., 2014; Royo et al., 2016) and roots from *V. vinifera* and *Vitis* rootstocks (Cookson et al., 2013; Corso et al., 2015; Cochetel et al., 2017; Livigni et al., 2019). Further studies investigated the plant responses to ozonated water applications (Campayo et al., 2021), the circadian cycle (Carbonell-Bejerano et al., 2014b; Rienth et al., 2014a), the interaction with abiotic stresses such as temperature (Liu et al., 2012; Carbonell-Bejerano et al., 2013; Xin et al., 2013; Rienth et al., 2014b; Rienth et al., 2016), light (Pontin et al., 2010; Carbonell-Bejerano et al., 2014a) and water availability (Perrone et al., 2012; Dal Santo et al., 2016b).

Regarding pathogens, grapevine is highly susceptible to a range of fungal diseases such as downy mildew (*Plasmopara viticola*) and powdery mildew (*Erysiphe necator*), and the bunch or noble rot (*Botrytis cinerea*). Therefore, several efforts have been made to study these interactions by performing transcriptomic analyses, although most of these have been performed on leaves (Fung et al., 2008; Polesani et al., 2010; Wu et al., 2010; Weng et al., 2014; Amrine et al., 2015; Li et al., 2015).

A few studies have used transcriptomics to better understand the impact of agronomic practices such as defoliation (Pastore et al., 2013; Zenoni et al., 2017) or cluster thinning (Pastore et al., 2011) on fruit quality, showing that these techniques, when applied at the appropriate phenological stage, can improve the quality of ripening fruits, in term of sugars and colors. Moreover, an increasing interest in the transcriptome profiles of other *Vitis* species has been asserted. A complete list of all transcriptomic grapevine studies in fruits is available in **Supplementary Table 1A**.

Thanks to the large number of public experiments, several transcriptome-related tools have been developed to explore this type of data. The Vitis Co-expression database ‘VTCdb’ (Wong et al., 2013) offered an online platform for exploring potential regulatory networks by addressing gene co-expression. The VTCdb was replaced by ‘VTC-Agg’ in Wong, 2020, by gene rank of correlations and aggregate networks, being constructed from 1,359 microarray samples (33 experiments). More recent applications include ‘AggGCN’, present in the VitViz platform (unpublished), which offers condition-dependent and independent aggregated networks from all SRA-located RNA-seq experiments, and ‘VESPUCCI’ (Moretto et al., 2016; Moretto et al., 2022), a cross-platform expression compendium that was carefully constructed by collecting, homogenizing, and re-annotating the metadata of publicly available microarray and RNA-Seq data (271 experiments). The GRape Expression Atlas ‘GREAT’ (unpublished) allows to query, visualize and analyze genes of interest with interactive heatmaps or expression tables by inferring public RNA-seq data (about 2,000 samples) from *Vitis vinifera*. Finally, ‘OneGenE’ (One Gene network Expansion) (Pilati et al., 2021) is a transcriptomic data mining tool that finds direct correlations between genes, thus producing association networks by using a causality-based method.

### Metabolomics

The use of plant metabolite profiling as a new tool for gene functional analyses and plant phenotyping has been incorporated in the last two decades (Fiehn et al., 2000). However, given the vast diversity of plant metabolites, metabolomic approaches are often based on separately analyzing different classes of metabolites having similar physical or chemical properties or functional groups. Hence metabolome studies are based on applying different analytical methods, including variable extraction protocols and instruments. A complete list of metabolomic studies in grapevine is available in **Supplementary Table 1B**.

The first metabolomic profiling study comprehensively reporting important grape secondary metabolites was achieved by Mattivi et al. (2006), where 91 grape cultivars were characterized for different polyphenols (focusing on the type and amount of flavonols and anthocyanins) at ripening on the berry skins. In particular and on average, the main flavonols found in red and white grapes differed in abundance order, and interestingly, the delphinidin-like flavonols were missing in all white cultivars, suggesting that the genes coding for flavonoid 3′,5′-hydroxylases were not expressed in these cases. A further study identified a hundred grape polyphenols by UHPLC/QTOF, providing a compendium of grape flavonols, anthocyanins, and stilbenes (Flamini et al., 2015). Other studies examined anthocyanin profiles in berry skins during the progress of ripening (Castellarin et al., 2006), the polyphenolic composition of PIWI grape cultivars (from the German fungus-resistant cv. ‘Pilzwiderstandsfähige’) (Ehrhardt et al., 2014; Gratl et al., 2021), or of wild American genotypes (Narduzzi et al., 2015; Ruocco et al., 2017). Cultivar-specific compositions of polyphenols have also been assessed, for instance, in ‘Muscat’ cultivars with anthocyanin profiles driving the main divergence between red and white cultivars, followed by flavonols and flavanols discriminating among the white accessions (Degu et al., 2015). Additionally, the responses to several stresses (Degu et al., 2016), the changes in the flavonoid profiles by early mechanical leaf removal (VanderWeide et al., 2018), or the stimulatory effect of kaolin application in leaves (Conde et al., 2016) have also been accomplished. Metabolomic analyses have also been performed to compare the primary metabolites of fruits (Dai et al., 2013; Cuadros-Inostroza et al., 2016; Zhao et al., 2016) and leaves (Harb et al., 2015; Conde et al., 2018). Other studies have focused on the analysis of the volatile compounds in roots (Lawo et al., 2011), leaves (Weingart et al., 2012), and berries (Vrhovsek et al., 2014).

Research has also been carried out to study the berry late-ripening program in skin, flesh, and seed (Vondras et al., 2017) or to understand the role of drought conditions affecting several metabolic pathways (Hochberg et al., 2013; Griesser et al., 2015; Hochberg et al., 2015b; Herrera et al., 2017; Pinasseau et al., 2017). In addition, the effect of temperature (Hochberg et al., 2015a) and light (Reshef et al., 2017; Reshef et al., 2018) have also been investigated.

Like for transcriptomics, metabolomic approaches have also been deployed to understand plant-pathogen interactions. For example, the identification of biomarkers of defense response to *Plasmopara viticola* has been a trending topic (Adrian et al., 2017; Chitarrini et al., 2017; Negrel et al., 2018; Ciubotaru et al., 2021), as well as the action of *Botrytis cinerea* on the fruit metabolism (Hong et al., 2012; Negri et al., 2017).

In recent years, metabolomics in grapevine has been coupled to transcriptome analyses to understand in detail the physiological mechanisms of berry ripening and the interaction with biotic or abiotic stresses. This topic will be further discussed later on.

### Proteomics

Approximately one-hundred grapevine proteomic studies can be found in the literature (a complete list of proteomic studies in grapevine is available in **Supplementary Table 1C**). The first extensive study on grapevine proteomics analyzed the mesocarp (flesh)-allocated proteins of ripe berries in six different cultivars using two-dimensional electrophoresis followed by MALDI-TOF peptide mass fingerprinting (Sarry et al., 2004). The authors determined the composition of 67 major proteins in ripe fruits and provided new evidence on the metabolism of sugar and organic acids in fruits. A second extensive work studied the proteomic dynamic changes during berry development and ripening in the mesocarp of cv. ‘Nebbiolo’ by profiling 101 proteins over seven time points (Giribaldi et al., 2007). The authors found that the majority of proteins were linked to metabolism, energy and protein synthesis and fate. In the same year, another study investigated the impact of two major abiotic stresses, water deficit and salt stress, on the shoot proteome of two cultivars, cv. ‘Chardonnay’ and cv. ‘Cabernet Sauvignon’, showing that the protein concentration varied mainly in response to the cultivars, then with time, and lastly with the abiotic stress (Vincent et al., 2007).

The knowledge of the grapevine berry proteome has been improved over the years by different studies focusing on skin proteome dynamics throughout berry ripening (Deytieux et al., 2007; Negri et al., 2008a; Martínez-Esteso et al., 2011a), on the identification of the different proteins present in skin, flesh or seed (Tian et al., 2015), on plasma membrane protein expression either during berry ripening (Zhang et al., 2008) or at maturity (Negri et al., 2008b), on the changes of the vacuolar proteome during ripening (Kuang et al., 2019), on single-berry proteome during development and ripening (Martínez-Esteso et al., 2011b; Martínez-Esteso et al., 2013) or in the protein expression profiles of grape berry during postharvest withering process (Di Carli et al., 2011).

Proteomics has also been employed to identify proteins associated with flavor volatile compounds found in fruits, such as proteins involved in the phenylpropanoid pathway, terpene synthesis, fatty acid derived volatiles and esters (Kambiranda et al., 2016; Kambiranda et al., 2018), or with the effects of ABA treatments on ripening *Vitis vinifera* berries (Giribaldi et al., 2010), sunlight exposure (Niu et al., 2013) and water deficit (Cramer et al., 2013). In addition, many other studies have tested proteome changes in plant-pathogen responses. For example, several studies investigated the leaf response to biotic stress, such as *Plasmopara viticola* (Milli et al., 2012; Figueiredo et al., 2017; Nascimento-Gavioli et al., 2017; Santos et al., 2020; Liu et al., 2021), while another study identified potential protein markers in berries affected by noble rot (Lorenzini et al., 2015).

### Ionomics

So far, only a few ionomic studies have been performed in grapevine. In 2011 the first ionomic work was published, describing the accumulation pattern of 42 mineral elements in cv. ‘Chardonnay’ berries during development and ripening (Bertoldi et al., 2011). The authors described that seven elements accumulate prior to veraison, other eighteen accumulate mainly prior to veraison but also during ripening, and seventeen progressively during growth and ripening. With regard to distribution, eight, sixteen and eighteen elements specifically accumulated in seeds, skin and flesh, respectively. In another study, the concentration of 34 mineral elements in grapevine berries was determined by ICP-MS in cv. ‘Corvina’ berries, harvested from eleven vineyards, to trace their geographical origin (Pii et al., 2017). Moreover, the analysis of cations such as K^+^, Mg^2+^, Ca^2+^, ${\text{NH}}_{4}^{+}$ in berries at different developmental stages showed the diversity of their concentration in several cultivars, providing additional information for the selection of genotypes able to cope with the adverse effects of climate change on fruit quality (Bigard et al., 2020). Finally, the ionomic signature was also studied in leaves identifying a mineral element-based response to *Xylella fastidiosa* (De La Fuente et al., 2013) and the changes in mineral distribution after *Plasmopara viticola* infection (Cesco et al., 2020). A complete list of ionomic grapevine studies is available in **Supplementary Table 1D**.

## Multi-omics integration of transcriptomics and metabolomics

In addition to single-omic studies, integrative omics analyses can be applied to provide a deeper understanding of the regulatory processes controlling different molecular phenotypes. To date, around sixty multi-omic studies combine transcriptomics and metabolomics, to study either the early and late responses to abiotic stresses, such as water and salinity stress (Cramer et al., 2007), the molecular mechanisms involved in berry development (Deluc et al., 2007; Fortes et al., 2011; Degu et al., 2014; Fasoli et al., 2018; Griesser et al., 2020) and over-ripening (i.e., post-harvest withering; Zenoni et al., 2016; Zenoni et al., 2020), or the changes in polyphenol and aromatic compound content in ripening berries (Costantini et al., 2015; Malacarne et al., 2015; Costantini et al., 2017). Other integrated approaches have studied berry responses during ripening to water deficit (Deluc et al., 2009; Savoi et al., 2016; Savoi et al., 2017; Yang et al., 2020), light (Suzuki et al., 2015; Sun et al., 2019; He et al., 2020; Zhang K et al., 2021) or temperature (Lecourieux et al., 2017). A few additional studies have also studied the ‘genotype by environment’ (GxE) interaction (i.e., the effect of terroir) on the plasticity of red and white grapes (Dal Santo et al., 2013; Anesi et al., 2015; Dal Santo et al., 2016a; Dal Santo et al., 2018).

The integration of transcript and metabolite data has contributed to the biological understanding of grape cultivar differences for fruit composition. For example, Degu et al. (2014) identified a cultivar-dependent regulation of specialized metabolism towards fruit maturation when comparing the cv. ‘Shiraz’ and ‘Cabernet Sauvignon’, the former displaying a higher upregulation of the entire polyphenol pathway, favoring the accumulation of piceid and *p*-coumaroylated anthocyanins. Moreover, in a post-harvest withering study, the expression of stilbene biosynthesis genes increased after harvest in a genotype-dependent manner, matching the varietal accumulation differences (Zenoni et al., 2016).

The integration of multi-omic datasets has been helpful in the identification of putative candidate genes regulating the accumulation of secondary metabolites in fruits. In this regard, a nudix hydrolase was identified as a component of a terpene synthase-independent pathway, enhancing monoterpene biosynthesis together with other genes potentially involved in terpenoid metabolism, such as cytochrome P450 hydroxylases, epoxide hydrolases, and glucosyltransferases (Costantini et al., 2015). Transcript and metabolite analyses determined that the biosynthesis of anthocyanins was a consistent hallmark of noble rot in white-skinned cv. ‘Sémillon’ (Blanco-Ulate et al., 2015), an unexpected response due to the absence of functional alleles of the MYBA1-A2 anthocyanin-regulators. This phenomenon led to the hypothesis of novel regulators controlling berry skin pigment production, which were later found by Matus et al. (2017) and D’Incà et al. (2021). The use of metabolomics (stilbenoid-oriented) and transcriptomics, coupled to the genome-wide exploration of transcription factor binding sites through DAP-seq, allowed Orduña et al. (2022) to identify new candidate genes coding for resveratrol modifying enzymes including laccasses, glycosyltransferases and *O*-methyl-transferases, potentially producing viniferin, piceid and pterostilbene, respectively. Finally, Savoi et al. (2016) associated the up-regulation of the *MYB24* transcription factor with the observed increased biosynthesis of three key monoterpenes under water deficit in white grapes, leading to the hypothesis of its capacity to regulate terpene synthase (*TPS*) gene expression. Indeed, this MYB24-*TPS* regulatory relationship was recently confirmed by Zhang C et al. (2021), who also integrated several omics including transcriptomics, metabolomics, and DAP-seq.

Numerous works have applied integrative methods to address responses to pathogen infection, most being conducted in fruits or leaves. For example, the leaf analysis of ‘Regent’ and ‘Trincadeira’ cultivars, respectively resistant and susceptible to mildew, has provided information on the different metabolic pathways related to the defense process (Figueiredo et al., 2008). The work of Malacarne et al. (2011) combined metabolic and transcriptional profiles in a segregating population for resistance to *Plasmopara viticola*, while Maia et al. (2020) suggested several gene/metabolite biomarkers of fungal/oomycetes-associated disease susceptibility in eleven *Vitis* genotypes. In addition, combined approaches have been employed to study berries infected with the fungus *Botrytis cinerea* (Agudelo-Romero et al., 2015; Blanco-Ulate et al., 2015), and also to determine how the interaction with this same pathogen at flowering influences quiescence and egressed infection (Haile et al., 2017; Haile et al., 2020). Recently, a few studies have examined the defense responses in susceptible and resistant grape cultivars (Eisenmann et al., 2019; Chitarrini et al., 2020), while others have examined the transcriptional, hormonal, and metabolic changes under powdery mildew infection caused by *Erysiphe necator* in leaves and berries (Pagliarani et al., 2020; Pimentel et al., 2021).

To date, only three publications integrate transcriptomics, proteomics, and metabolomics in a single study. By overlapping genes, proteins, and metabolites, Zamboni et al. (2010) identified stage-specific biomarkers for berry development and withering. Moreover, multi-omic datasets were integrated to test the uniqueness of three red-skinned and two white-skinned cultivars at berry maturity, providing a detailed indication of genotype differences (Ghan et al., 2015). Finally, multi-omic analyses have been employed in the study of leaf responses to copper stress, providing agronomic knowledge to improve vineyard management and favor the breeding of copper-resistant grape cultivars (Chen et al., 2021).

## An illustration of data integration and meta-analysis using the TransMetaDb app

Moving forward from single-omic studies and *gene-to-gene* network exploring tools, the construction of *gene*-*to*-*metabolite* networks *via* integrative approaches represents a promising strategy for identifying novel gene functions and unprecedent links between genes and metabolites (Hirai et al., 2005; Saito et al., 2008). This concept has been reviewed in grapevine (Wong and Matus, 2017; Matus et al., 2019), stating the need for appropriate tools to visualize the integration of multi-omic datasets. The first tool developed for data integration in grapevine was ‘VitisNet’ (Grimplet et al., 2009; Grimplet et al., 2012), which allowed the multiple combinations of omics data by overlapping user data to 247 curated grapevine molecular networks, reporting more than 16,000 genes; unfortunately, this tool is no longer accessible. A second developed tool was ‘VitisCyc’ (Naithani et al., 2014), a grape-specific database for browsing and visualizing metabolic pathways end enzymatic reactions, compounds, genes and proteins, and for comparing metabolic networks with other publicly-available resources from other plant species (this tool is now hosted in the Gramene database; Tello-Ruiz et al., 2021).

**Figure 2:**
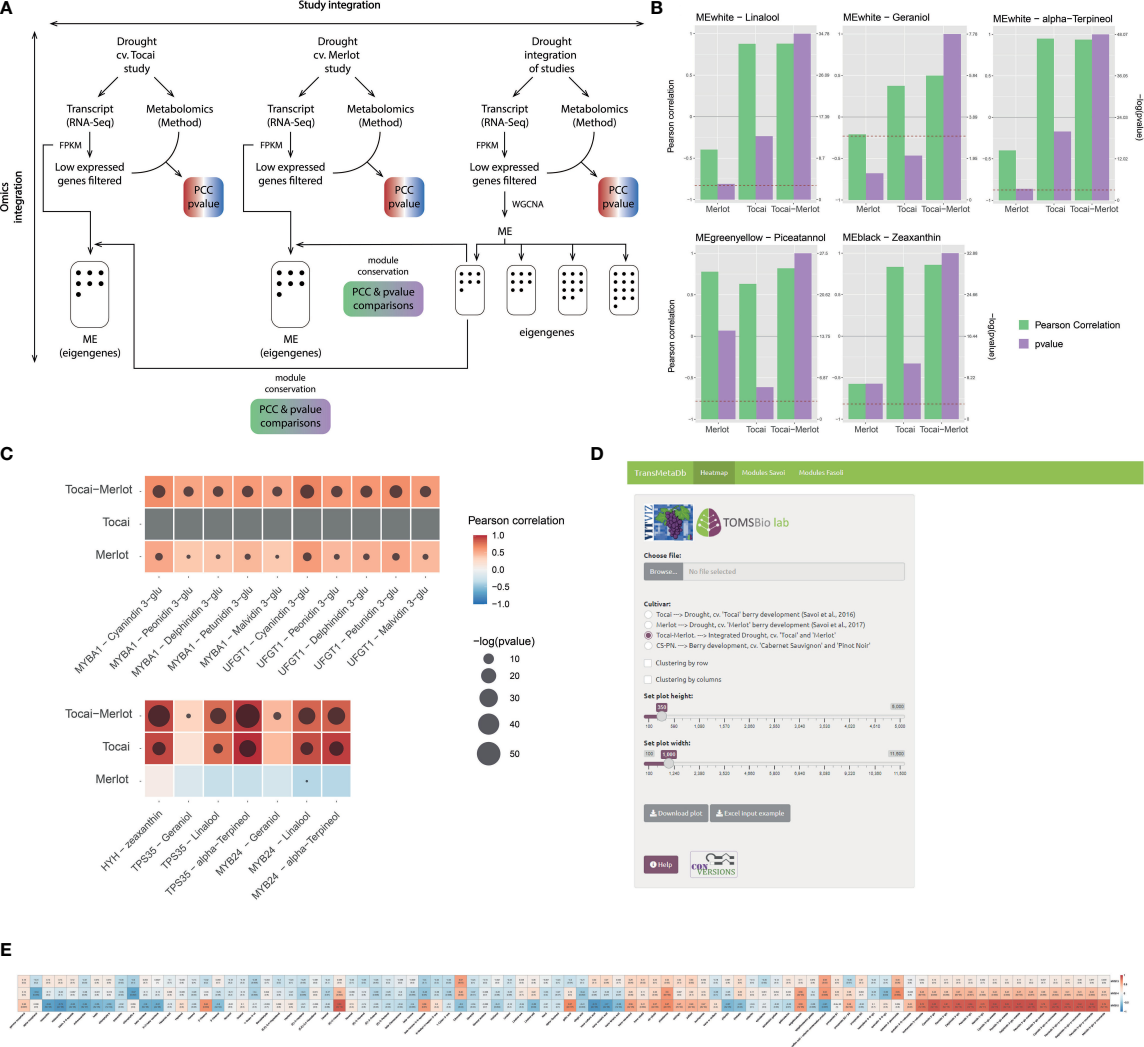
Omics integration in two studies that investigated the effect of a long-lasting water deficit on the metabolism of white (cv. ‘Tocai friulano’) and red (cv. ‘Merlot’) cultivars during berry development and ripening (Savoi et al., 2016; 2017). **(A)** Methods scheme of the analyses performed for each of the independent studies and the combination of them (i.e., study integration or meta-analysis). Genes with low expression are filtered-out before WGCNA analysis. **(B, C)** Results obtained from the analyses depicted in **(A)**, where **(B)** Improvement of statistical significance and/or correlation between genes and metabolites for the combination of both studies. P-value representation is only shown when the value exceeds the 0.05 threshold. **(C)** Improvement of statistical significance and/or correlation between gene modules and metabolites for the combination of both studies. A −log_10(_p-value) scale is provided where a higher value represents a greater statistical significance. The red dashed line depicts −log_10(_p-value) corresponding to a 0.05 threshold. **(D)** Visual interface of the TransMetaDb App, found at the *Vitis* Visualization (VitViz) platform (https://tomsbiolab.com/vitviz). The resulting example heatmap is shown in **(E)**.

To date, there is no visualization tool in grape to explore the correlation of transcriptomics and metabolomics data, and even less to combine them in meta-analyses. Within the COST Action CA17111 INTEGRAPE, we have developed TransMetaDb (**Figure 2**), an application available in the *Vitis Visualization* (VitViz) platform (https://tomsbiolab.com/vitviz), to freely explore the correlation between metabolites and genes in multiple transcriptomics/metabolomics integrated datasets. The Integrated Transcriptomics and Metabolomics Database Application (TransMetaDb) was developed in the *Shiny R* environment and offers a user-friendly interface to explore transcriptomic data (i.e., differentially expressed genes and co-expression modules) and its correlation to metabolites quantified in the same conditions. The App takes an Excel or comma/tab-separated file as an input, with only one column containing the 12X.v2 VCost.v3 gene IDs of interest, and an optional second column with the gene names/symbols, whose purpose is to provide a descriptive name for the user-provided genes. The output is a downloadable heatmap or table, with all the genes of interest by row, compared with the different metabolites on each column. Each cell contains the Pearson’s correlation coefficient (PCC) value and its *p*-value in brackets, calculated using the student asymptotic method. The identification of gene candidates related to metabolite phenotypic traits has often been inferred from association measures, such as the commonly-used PCC method (to measure linear correlation between two variables). Although alternative measures can be employed, PCC is still the standard for initial exploration of *gene-to-gene* and *gene-to-metabolite* networks (Nikiforova et al., 2005; Mao et al., 2009). This is a first general approach to discover functions in a set of genes and metabolites of interest. On a second tab, the App allows the user to explore co-expression clustered modules, downloading a table with all gene-module categories.

So far, three studies have been uploaded to the App; Savoi et al. (2016) and Savoi et al. (2017) focused on the impact of drought on fruit secondary metabolism and have a similar experimental design, the same metabolomic method, with all data being publicly available. A third study detailed a transcriptomic and metabolomic temporal map of berry development in two cultivars for three consecutive years (Fasoli et al., 2018). This data was first reanalyzed; briefly, the transcriptomic data was re-aligned using STAR v. 2.7.3a (Dobin et al., 2013) against the PN40024 12X.v2 assembly, and a raw gene count matrix was extracted using the latest VCost.v3 annotation (Canaguier et al., 2017) and *featureCounts* (Liao et al., 2014). Gene expression was normalized with FPKM (Fragments per kilobase per million mapped reads) as this normalization method takes into account both sequencing depth of libraries and gene length, allowing the comparison of expression between different genes across different samples (other normalization methods will be applied in the future, such as TMM).

Clustering of gene expression and metabolite profiles can serve for mining useful information from noisy data, identifying cohorts of genes that control metabolism and understanding how metabolic pathways can be rewired in development and stress. Detection of functional gene modules by classical clustering methods, such as *K-*means or mean-shift, have been outperformed in transcriptomic studies by a modification of the algorithm, e.g., the fuzzy *k-*means applied in the *fclust* R package (Ferraro et al., 2019), or by correlation networks using for instance the Weighted Gene Correlation Network Analysis (WGCNA) R package (Langfelder and Horvath, 2008). In TransMetaDb, genes have been clustered in eigen modules (ME) by applying WGCNA, and metabolites have been associated to these modules in the same way as for genes. As explained in Langfelder and Horvath (2008), WGCNA works by generating a network (or adjacency matrix) from the biological data and then performing a hierarchical clustering. In order to build the adjacency matrix, an intermediate co-expression similarity matrix is defined using the biological data by computing co-expression measures between genes. Once the co-expression similarity matrix is constructed, it is transformed in the adjacency matrix using a beta power value that is chosen by the user in order to generate an adjacency matrix, and that satisfies the scale-free network topology where the distribution of node degree is adjusted to a potential law (i.e., high number of nodes with a low number of edges and few nodes with a high number of edges). For doing this analysis, the blockwiseModules function was used with a deepSplit of 4, a mergeCutHeight of 0.1 and a beta power of 30 for the integration of the ‘Tocai friulano’ and ‘Merlot’ studies and a power of 11, deepSplit of 4 and a mergeCutHeight of 0.2 for Fasoli et al. (2018). Following *WGCNA* authors’ recommendation, the deepSplit and mergeCutHeight parameters were adapted to obtain a number of eigen modules and a number of genes per eigen module adaptable for subsequent analysis with a minimum module size of 40 genes. Transcripts and metabolites were integrated in gene-*to*-metabolite and eigen module-*to*-metabolite matrices for each of the individual and combined studies.

While exploring the WGCNA results of the combined studies we observed that the Pearson correlation coefficients (PCC) and/or the significance (*p*-values) of specific eigen module (ME, **Figure 2B**) or gene-metabolite (**Figure 2C**), often increased. This is expected, because as the sample is larger the error is lower and the sample correlation converges to the population parameter, in the same way as increasing the number of replicates improves the inferences that can be made about a population. For example, in the combined eigen module “white”, PCC and *p*-values of linalool, geraniol, and α-terpineol were higher and more significant in the ‘Tocai friulano’+’Merlot’ set, just like the association between these same three terpenes (present in the white cultivar only) and the genes *MYB24* and *TPS35* (Savoi et al., 2016; Zhang C et al., 2021). Moreover, the addition of an anthocyanin-free dataset (i.e., Tocai) to the data belonging to an anthocyanin-pigmented cultivar (i.e., Merlot) also improved the transcript-metabolite correlations between different pigments and related genes as the ‘no expression’ and ‘no pigment accumulation’ is a positive correlation in itself. The five glycosylated anthocyanins (only present in the red cultivar) and the anthocyanin-related genes *UFGT* (Boss et al., 1996) and *MYBA1* (Kobayashi et al., 2004) showed increased correlation and/or increased significance in the meta-analysis compared to individual studies (where the correlation was already high). This improvement is due to the coherent behavior of the selected proteins as they correspond to the latest enzyme of the pathway and the master regulator of anthocyanin accumulation, respectively, representing a perfect case of expected correlation. Finally, we observed the same trend for the light-induced carotenoid zeaxanthin and the light/UV signaling gene *HYH* (HY5 HOMOLOGUE; (Loyola et al., 2016). These results clearly imply that meta-analyses of integrated transcriptomic/metabolomic datasets improves the strength of correlation metrics in the same way as adding new samples in the construction of a gene co-expression network increases the network performance until it reaches a plateau (Wong, 2020).

We plan to increase the number of combined transcriptomic and metabolomic datasets in TransMeta Db, based on the quality of the metadata. Metabolic data will be compared in the following submitted studies to see which metabolites can be combined. If this data has been acquired by different platforms, or if it is presented in different units, one possibility would be to scale the data (e.g., Z-score) before integration. Nevertheless, this effort would certainly push the community to improve the annotation of their experiments in public repositories so their data can be fully interoperable. Once more studies have been uploaded, we also expect to showcase alternative correlation metrics using other compatible software and pipelines.

## Data availability statement

The datasets presented in this study can be found in online repositories. The names of the repository/repositories and accession number(s) can be found in the article/**Supplementary Material**.

## Author contributions

SS and JM conceived the study and wrote the manuscript. AS and LO contributed with the reanalysis of the transcriptomic and metabolomic data set and by building the TransMetaDb app found in the VitViz platform. All authors contributed to the article and approved the submitted version.

## Funding

This publication is based upon work from COST Action CA17111 INTEGRAPE, supported by COST (European Cooperation in Science and Technology) and COST grant ECOST-STSM-Request-CA17111-48997 awarded to SS. This work was also supported by Grants PGC2018-099449-A-I00, PID2021-128865NB-I00 and by the Ramón y Cajal program grant RYC-2017-23645, all awarded to JM, and to the FPI scholarship PRE2019-088044 granted to LO from the Ministerio de Ciencia, Innovación y Universidades (MCIU, Spain), Agencia Estatal de Investigación (AEI, Spain), and Fondo Europeo de Desarrollo Regional (FEDER, European Union).

## Acknowledgments

We thank Anne-Marie Digby (University of Verona) for manuscript revision. We apologize to all authors whose work has not been cited owing to space constraints.

## Conflict of interest

The authors declare that the research was conducted in the absence of any commercial or financial relationships that could be construed as a potential conflict of interest.

## Publisher’s note

All claims expressed in this article are solely those of the authors and do not necessarily represent those of their affiliated organizations, or those of the publisher, the editors and the reviewers. Any product that may be evaluated in this article, or claim that may be made by its manufacturer, is not guaranteed or endorsed by the publisher.

## Supplementary material

Supplementary Table 1 List of **(A)** transcriptomics, **(B)** metabolomics, **(C)** proteomics, and **(D)** ionomics studies retrieved from the literature. Data was collected from NCBI using different queries and keywords. Only the studies published until 12/2021 were considered. Data were manually curated to remove clear outliers.
